# Reducing zero-dose children and improving routine immunization following COVID-19 through community health workers in Cameroon

**DOI:** 10.11604/pamj.2026.53.160.48816

**Published:** 2026-04-15

**Authors:** Martha Ndiko Ngoe, Pamela Besong Oben, Daniel Nebongo Nebongo, Perpetua Bihnwi Tayoh Yelluma, Wilson Fosack Nsanda, Margaret Watkins

**Affiliations:** 1 Expanded Programme on Immunization, South West Regional Delegation of Public Health, Buea, Cameroon,; 2 Expanded Programme on Immunization, Central Coordination, Yaoundé, Cameroon,; 3 District Health Service Kumba North, Kumba, Cameroon,; 4 District Health Service Kumba South, Kumba, Cameroon,; 5 The Sabin Vaccine Institute, Washington DC, United States of America

**Keywords:** Conflict-afflicted, community health workers, zero-dose children, missed opportunity for vaccination

## Abstract

Routine immunization in Cameroon was severely disrupted by the COVID-19 pandemic, compounding longstanding challenges stemming from sociopolitical instability in the South-West Region. Kumba, hosting large populations of internally displaced persons (IDPs), reported increasing numbers of zero-dose and under-immunized children. This study assessed the effect of training Community Health Workers (CHWs) and Health Care Workers (HCWs) to identify, refer, and link zero-dose and under-vaccinated children to services in Kumba North and South Health Districts. Six low-performing health areas were selected based on routine immunization data. From October 2022 to February 2023, CHWs conducted household visits, identified unvaccinated children, and provided referrals, while HCWs received training on reducing missed opportunities for vaccination (MOV). Monthly supervision and data review supported implementation. Due to large, mobile IDP populations, outcome measures focused on doses administered, not coverage. Costs were estimated using operational expenditures and UNICEF vaccine procurement prices. Penta-1 doses increased from 285 pre-intervention to 3,172, while Penta-3 doses increased from 321 to 2,893. HPV vaccination reached 2,134 adolescents (1,747 girls; 387 boys). CHWs conducted 945 home visits and identified 299 zero-dose children. Operational cost was USD 7,240, and the estimated value of vaccines administered was USD 31,101. Although external factors such as fluctuating conflict intensity or sporadic outreach may have contributed, these were not consistently implemented in the intervention areas. CHW engagement combined with HCW MOV-reduction training substantially increased doses administered in a conflict-affected setting. This low-cost, scalable model can strengthen routine immunization and reduce zero-dose inequities in humanitarian contexts.

## Introduction

The COVID-19 pandemic caused major disruptions to essential health services globally, with routine immunization systems among the most affected [1,2]. In Cameroon, these disruptions exacerbated underlying health system weaknesses and overlapped with the sociopolitical crisis affecting the Northwest and Southwest Regions. Since 2015, protracted armed conflict has resulted in widespread displacement, limited access to health facilities, and inconsistent service delivery [3,4].

Kumba, a key urban centre in the Southwest Region, hosts substantial numbers of internally displaced persons (IDPs) fleeing violence from surrounding rural areas. Displacement, insecurity, and the closure or destruction of health facilities have led to pockets of zero-dose children—those who have never received routine vaccinations—and under-immunized children who remain vulnerable to measles, polio, and other vaccine-preventable diseases [5]. Vaccine misinformation and fears associated with COVID-19 further reduced healthcare-seeking behaviour, worsening declines in immunization coverage [4,6]. These factors threatened to erode decades of progress in immunization coverage, especially in fragile settings where health systems were already overstretched [2].

Community Health Workers (CHWs) are recognised as essential in extending primary healthcare services to underserved populations, especially in fragile and hard-to-reach settings [7]. In Cameroon, however, CHWs are primarily engaged during mass campaigns and are not routinely integrated into continuous immunization activities, limiting their potential to support sustained outreach and follow-up [8]. In this context, integrated community- and facility-based strategies are needed to rebuild routine immunization performance. This study evaluated an intervention that trained and deployed CHWs to identify and refer zero-dose and under-immunized children, alongside training for health care workers (HCWs) to reduce missed opportunities for vaccination (MOV). Implemented in Kumba North and South Health Districts, the intervention sought to improve vaccination uptake amid conflict-related disruptions and the lingering effects of the COVID-19 pandemic [5,9,10].

## Methods

**Figure 1:**
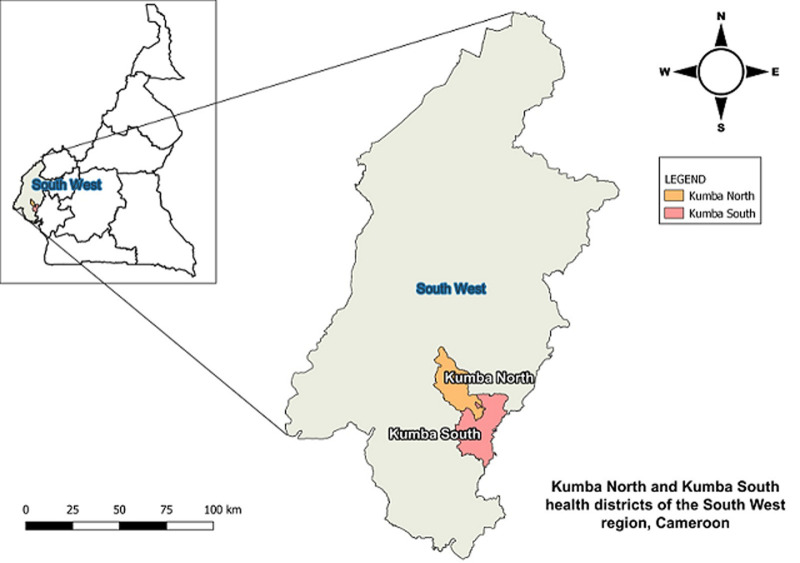
geographic location of the areas of implementation for the community Health Workers intervention: Kumba North and Kumba South Health Districts in the Southwest Region of Cameroon

**Study design and setting:** this implementation study was conducted in the Kumba North and Kumba South Health Districts of Cameroon's Southwest Region—areas heavily affected by sociopolitical instability since 2015. The conflict has caused mass displacement, intermittent insecurity, and significant disruptions to routine health services [3,5]. Kumba serves as a refuge for large numbers of IDPs, many of whom face barriers to accessing immunization services. The combined population is estimated at 400,000, with a high proportion of children under five. Routine immunization performance has been hindered by insecurity, population mobility, occasional stock-outs, reduced health-worker availability, and widespread COVID-19-related vaccine misinformation. These contextual challenges necessitated a community-centred strategy [6] (Figure 1).

**Pre-implementation assessment:** a comprehensive review of routine immunization data from 2020 and 2021 was undertaken to establish baseline vaccination coverage and identify health areas most affected by low immunization rates. Data from the District Health Information System (DHIS2) and monthly reports from health facilities were analysed to detect trends in vaccine uptake and to identify priority intervention sites. Thirteen health areas were present across the two districts; six were prioritized based on indicators such as low pentavalent vaccine uptake, security and accessibility constraints, and high IDP concentrations. Selected areas included a mix of urban, peri-urban, and rural communities, providing a diverse context for implementing the intervention.

**Stakeholder engagement and training:** engagement meetings were conducted with the Ministry of Public Health's Expanded Programme on Immunization (EPI) Southwest Office, district health teams, community leaders, and IDP representatives to ensure ownership and alignment with national immunization strategies. Training was conducted for 26 HCWs, 10 CHWs, and 2 CHW supervisors. Topics included: identification and referral of zero-dose and under-immunized children; community mobilisation; interpersonal communication; vaccine misinformation; cold-chain basics; data recording and reporting; and MOV-reduction strategies. Ten CHWs participated in field work and received stipends; two served as supervisors.

**Implementation activities:** from October 2022 through February 2023, CHWs conducted routine home visits within their assigned communities to identify zero-dose and under-vaccinated children. Using standardized screening tools, CHWs assessed vaccination status by reviewing vaccination cards or caregiver recall and referred eligible children to fixed health facilities or outreach posts for vaccination. CHWs also provided health education on the importance of immunization and addressed concerns related to vaccine safety and efficacy. Community volunteers were selected and trained to act as CHW supervisors to support daily field activities, provide technical guidance, and monitor adherence to protocols. Integration of CHWs within the health system was facilitated by linking each CHW with a designated focal person at the nearest health facility, who provided technical support and facilitated data validation. Three rounds of supportive supervision visits were conducted during the intervention period by district health management teams. These visits were often integrated with other ongoing health programs to optimize resources and provide holistic support. Monthly data review meetings at the district level involved health area focal persons, CHW supervisors, and other stakeholders to track progress, validate data, address challenges, and provide feedback for continuous quality improvement.

**Outcome measures and data analysis:** because Kumba hosts large and fluid internally displaced populations, accurate denominators for the target population could not be determined. This prevented the calculation of precise immunization coverage. Therefore, outcome measures focused exclusively on doses administered, including: the number of zero-dose children receiving Penta-1; the number of children receiving Penta-3; the number of adolescents receiving HPV (by sex); the number of household visits conducted; and the number of CHWs, HCWs, and supervisors trained. All comparative results represent increases in doses administered, not population coverage. Quantitative data were collected through CHW registers and facility vaccination records. Descriptive statistics were used to summarise vaccination coverage before and after the intervention. Comparisons were made using percentage increases in coverage rates. Qualitative observations from supervisory reports and stakeholder feedback were incorporated to contextualize quantitative findings.

## Results

**Operational and vaccine costs:** the total operational cost for the five-month intervention amounted to USD 7,240. This covered essential expenses such as community health worker (CHW) stipends, transportation, supervision, training, community engagement, and data review meetings. In terms of vaccine costs, based on UNICEF procurement prices, the value of vaccines administered during this period was estimated at USD 31,101. This included USD 11,895 for Penta and USD 19,206 for the HPV vaccine.

**Pre-intervention immunization performance:** baseline data from 2020-2021 indicated a low uptake of routine immunization antigens, with high numbers of zero-dose and under-immunized children. Notably, HPV vaccination coverage among boys was not recorded before 2023, as the national rollout of the HPV vaccine for boys commenced in early 2023 (Table 1).

**Table 1:** pre-intervention routine immunization coverage (Kumba North and South Health Districts, Baseline)

District	Penta-1	Penta-3	HPV (Girls)	HPV (Boys)
Kumba North	151	212	549	0
Kumba South	134	109	397	0
Total	285	321	946	0

HPV = Human Papillomavirus; Penta-1 = First dose of pentavalent vaccine; Penta-3 = Third dose of pentavalent vaccine. Data collected from Kumba North and South health district routine immunization registers for 2020-2021.

**Post-intervention outcomes:** the intervention demonstrated significant improvements in vaccination uptake, though due to Kumba's highly mobile IDP population, it was not possible to obtain accurate population denominators. As such, the outcomes reflect the total number of vaccine doses administered, rather than population coverage. Between October 2022 and February 2023, several key outcomes were observed. The number of Penta-1 doses administered saw a dramatic increase from 285 to 3,172, and similarly, the number of Penta-3 doses increased from 321 to 2,893. These increases are indicative of a substantial rise in vaccine delivery, although it is important to reiterate that they refer to the total number of doses administered, rather than an increase in overall population coverage.

In terms of HPV vaccination, the intervention marked a notable achievement, as 2,134 adolescents were vaccinated. Among these, 1,747 were girls, and 387 were boys. This represented the first recorded doses of the HPV vaccine among boys in both districts, which aligns with the recent national introduction of the HPV vaccine for boys in early 2023. This shift underscores the successful expansion of vaccine access to adolescent boys, a critical step towards gender-equitable health interventions.

Community health workers (CHWs) played a crucial role in the intervention's success. Over the course of the intervention, they conducted 945 household visits. These visits were instrumental in identifying and referring 299 zero-dose children—those who had not yet received any routine vaccinations. This targeted approach was vital in reaching the most vulnerable children in the community, particularly in a setting where accurate denominators were unavailable due to the fluid nature of the IDP population. These outcomes highlight the success of the CHW-led outreach strategy and its potential for increasing vaccine coverage, particularly in difficult-to-reach populations. By engaging directly with communities and identifying children who had missed vaccination opportunities, CHWs contributed to the observed increases in vaccine doses administered.

**Figure 2:**
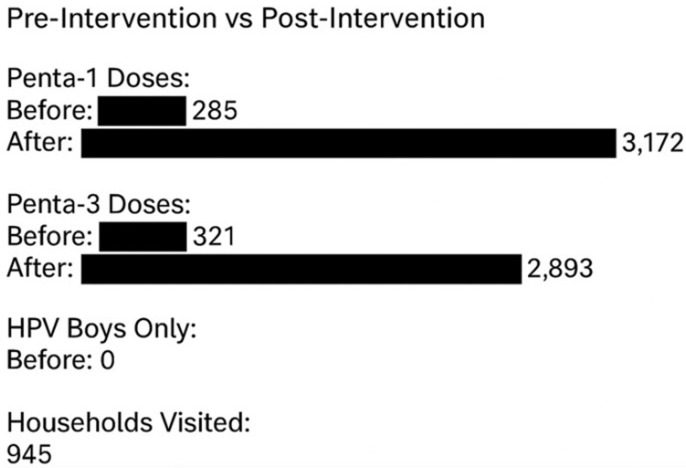
comparison of routine immunization coverage before and after community health workers intervention

**Qualitative findings:** qualitative findings from supervisory observations further contextualised and supported the quantitative results. Increased and sustained CHW presence within communities was associated with improved trust between caregivers and the health system, which facilitated greater acceptance of vaccination services. Health facility staff also demonstrated improved practices over the course of the intervention, particularly in client screening, vaccination documentation, and the reduction of missed opportunities for immunization. In addition, the intervention contributed to strengthened HPV-related communication, with clearer and more consistent messaging observed during community interactions. This improvement was reflected in increased acceptance of HPV vaccination not only among caregivers of girls but also among caregivers of boys, coinciding with the national expansion of HPV vaccination eligibility. Finally, routine monthly data review meetings enhanced data validation, accountability, and shared problem-solving among health workers and supervisors, contributing to overall improvements in programme performance. Routine immunization coverage before and after CHW intervention in the Kumba North and Kumba South Health Districts, Cameroon, shows the absolute numbers of changes for Penta-1 and Penta-3, the number of household visits, and the number of children tracked and referred for vaccination (Table 2) (Figure 2).

**Table 2:** post-intervention outcomes and activities (October 2022-February 2023)

Indicator	Number achieved
Zero-dose children vaccinated with Penta-1	3,172
Children vaccinated with Penta-3	2,893
Adolescents vaccinated with HPV (Girls)	1,747
Adolescents vaccinated with HPV (Boys)	387
Total HPV vaccinations	2,134
Household visits conducted by CHWs	945
Number of defaulters identified and referred	299
CHWs trained	6
HCWs trained	18
Community supervisors trained	4
Advocacy meetings held	3
Training workshops organized	2

Penta-1 and Penta-3 represent the first and third doses of the pentavalent vaccine; HPV = Human Papillomavirus; Zero-dose children (6 weeks old and above) are those who had not received any previous dose of Penta-1 vaccine, though eligible-data collected during the intervention implementation period (October 2022-February 2023) from routine district monitoring.

## Discussion

The findings of this study demonstrate that combining CHW-led outreach with HCW training focused on reducing missed opportunities for vaccination (MOV) can substantially increase vaccine uptake, even in conflict-affected environments. With a modest operational investment of USD 7,240, the intervention achieved significant gains in vaccination service utilization. These results align with existing evidence supporting the efficacy of community-based health interventions in fragile and conflict-affected settings [10,11].

While external factors such as fluctuating conflict intensity, sporadic government outreach activities, and intermittent NGO support could have influenced vaccination trends, several key points help contextualize the findings. First, no major vaccination campaigns were conducted in the six intervention health areas during the study period. Second, the fluctuations in conflict intensity were minimal, and they did not coincide with significant changes in health facility utilization. Furthermore, there were no new external funding sources, commodities, or partner-supported interventions introduced in the area during the study period. Given the consistency and magnitude of the observed improvements in vaccine doses administered, coupled with the documented activities of CHWs and strengthened MOV practices, it is reasonable to conclude that the intervention itself was the primary driver of these positive outcomes.

Due to the fluid nature of the IDP population in the area, accurate population denominators were not available, which prevented the calculation of immunization coverage rates. Consequently, all percentage changes presented refer solely to the increase in doses administered, rather than coverage. This distinction is crucial to avoid potential misinterpretation of the findings.

Several factors contributed to the success of the intervention. First, the efforts of CHWs in generating demand for vaccines played a key role. Through 945 household visits, CHWs identified and referred 299 zero-dose children, ensuring that hard-to-reach populations were targeted. Their proximity to the community allowed them to offer targeted counselling, address misinformation, and facilitate access to vaccination points, making the process more accessible for families. Second, the training provided to HCWs to reduce MOVs led to notable behavioural changes. Supervisory observations documented systematic vaccination status screening at every visit, vaccination of eligible children even if they were mildly ill or arriving late, and better documentation practices, including improved tracking of defaulters. The intervention also resulted in expanded use of outreach vaccination sessions and a reduction in the frequency of unnecessary deferrals. These improvements align with global evidence on MOV reduction, demonstrating the positive impact of targeted training on vaccination practices [12-15].

From a financial perspective, the operational cost of the intervention was modest when compared to the significant improvements observed in vaccination coverage. With an estimated USD 31,101 worth of vaccines administered, the intervention clearly demonstrated strong value for money, particularly given the high marginal impact of reaching zero-dose children. The per-child delivery cost was low relative to the improvements in vaccination uptake, further highlighting the cost-effectiveness of CHW-led outreach strategies in humanitarian settings [10].

Moreover, the success of the home visit strategy in identifying zero-dose children underscores the importance of decentralised, community-driven vaccination efforts [7,8,16]. Unlike traditional CHW engagement in Cameroon, which is primarily limited to mass campaigns, the routine and ongoing involvement of CHWs in this intervention enabled sustained outreach and demand generation [8].

Finally, strong stakeholder engagement, regular supervision, and monthly data reviews were essential enablers of the intervention's success. These factors ensured high implementation fidelity and accountability throughout the project, reinforcing the importance of maintaining structured oversight and active involvement from all relevant stakeholders to achieve sustainable impact [10,12].

**Limitations:** several limitations should be considered. The study lacked a control group, limiting causal inference of observed coverage gains solely to the intervention. Data were descriptive and did not adjust for dynamic population movements, such as displacement and migration, which are common in conflict-affected settings and could affect coverage estimates [3,5,6]. Additionally, sustainability remains uncertain: ongoing financing for CHWs and full integration into routine health systems beyond the project period present challenges [9,16].

## Conclusion

Training and deploying CHWs and HCWs markedly increased the number of doses administered to zero-dose and under-immunized children in conflict-affected districts [3,5,8]. CHW-led community engagement and MOV-reduction training for HCWs restored service utilisation following COVID-19 disruptions [2]. This model is adaptable, low-cost, and appropriate for humanitarian and fragile settings seeking to improve vaccine equity [11,15,17,18].
